# The efficacy and safety of Chinese massage in the treatment of acute mastitis: A protocol for systematic review and meta-analysis of randomized controlled trials

**DOI:** 10.1097/MD.0000000000028504

**Published:** 2022-01-21

**Authors:** Longsheng Ren, Jie Zhang, Ruiying Guo, Can Lyu, Danyang Zhao, Zhihao Dong, Zenglin He, Qiang Wang

**Affiliations:** a Shandong University of Traditional Chinese Medicine, Jinan, Shandong, China; b Shandong Provincial Hospital Affiliated to Shandong First Medical University, Jinan, Shandong, China.

**Keywords:** acute mastitis, Chinese massage, protocol, systematic review and meta-analysis

## Abstract

**Background:**

The incidence of acute mastitis (AM) in lactating women has been increasing year by year. If there is no timely and appropriate treatment, AM may develop into mammary abscess and septicemia. This special situation has aroused social attention. Chinese massage has been widely used in the treatment of AM in recent years, but there is no systematic review of the effect of Chinese massage on AM. We plan to explore the efficacy and safety of Chinese massage in the treatment of AM.

**Methods:**

We will use a computer to search the following 8 electronic databases (PubMed, Web of Science, Cochrane, Embase, Sinomed, China National Knowledge Infrastructure, Chongqing VIP Information, WanFang Data) on November 30, 2021. Randomized controlled trials (RCT) of Chinese massage therapy for AM were screened. Primary outcome measure: overall clinical response rate, breast pain score. Secondary outcome measures: milk secretion, temperature, mass size and time to resolution, White blood cell count, C-reactive protein, and the incidence of adverse reactions. According to the inclusion criteria and exclusion criteria, the included literature will be independently evaluated by two researchers using the RCT bias risk assessment tool in the Cochrane evaluation manual Handbook5.4, and meta-analysis will be performed by RevMan5.4 software. Funnel plots were used to analyze whether the study had publication bias.

**Results:**

We will evaluate the clinical effect of Chinese massage therapy on AM based on RCTs

**Conclusion:**

This study will provide evidence-based evidence for the effectiveness and safety of Chinese massage in the treatment of AM.

**Protocol registration number:**

INPLASY2021120019.

## 1 Introduction

Acute mastitis (AM) is an acute suppurative breast disease common in lactation women. According to the incidence survey of acute mastitis in lactating women, about 10% of women worldwide are affected by it.^[1,2]^ The main symptoms of AM are breast swelling and pain, breast lumps, and some patients have systemic manifestations such as red skin and elevated body temperature.^[3]^ Studies show that the main cause of acute mastitis is milk deposition and infection, and the main pathogenic bacteria is *Staphylococcus aureus*.^[4,5]^ Factors such as women's decreased physical resistance after childbirth, unhealthy dietary patterns, inappropriate breast-feeding methods and posture, and poor milk flow are considered to be important factors leading to the emergence of AM. ^[6]^ If AM cannot get timely and correct treatment, it will not only affect the baby's breastfeeding, but also lead to the occurrence of breast abscess, sepsis and other dangerous situations.^[7–9]^ Breastfeeding is the preferred method of feeding newborns up to six months of age, as advocated by the World Health Organization, and is beneficial to both mother and baby. Studies have shown that mastitis severely affects women who breastfeed, so timely and effective treatment of AM is especially important.^[10]^ At present, western medicine for AM in the early stage of the main treatment is anti-infection treatment, although large doses of antibiotics can achieve anti-inflammatory effect, reduce the relevant symptoms.^[11]^ However, the overuse of antibiotics affects the therapeutic effectiveness of AM and breastfeeding.^[12]^ If mammary abscess is present, the patient's recovery and the infant's feeding will be affected by the treatment. Therefore, how to safely and effectively treat patients with AM has become an urgent problem to be solved.

Chinese massage plays a role in dredging meridians, promoting qi and blood circulation, and enhancing the body's resistance to disease. In recent years, it has been reported that the effect of Chinese massage on acute mastitis is exact.^[13]^ Modern medicine believes that massage can improve the mammary gland blood circulation and lymph circulation, and can also promote the patency of mammary gland ducts. The problems of patients’ milk stasis and pain can be solved by Chinses massage.^[14]^ However, there has been no systematic review and meta-analysis of the effects of Chinese massage on AM. Based on the RCT, this study will evaluate the effectiveness and safety of Chinese massage in the treatment of AM by using meta-analysis, aiming to provide more evidence-based evidence for the clinical treatment of AM.

## 2 Methods

### 2.1 Study registration

Our protocol will be based on the preferred reporting items for systematic review and meta-analysis protocols (PRISMA-P) 2015.^[15]^

This systematic review and meta-analysis protocol has been registered on the INPLASY website (https://inplasy.com/). The registration number is INPLASY2021120019.

### 2.2 Ethics and dissemination

Our study was based on data from published RCTs, so ethical approval and informed patient consent were not required. Our findings will be published in a peer-reviewed journal.

### 2.3 Inclusion criteria for this study

#### 2.3.1 Types of studies

All of the studies included were RCTs of Chinese massage therapy for AM. All studies involving case reports, animal studies, reviews, basic research, and non-RCTs will be excluded.

#### 2.3.2 Types of participants

Patients meeting the diagnostic criteria for AM will be selected for our study. There are no restrictions on source, nationality, race, age, etc.

#### 2.3.3 Types of interventions

The control group only received conventional treatment of Western medicine. The treatment group was given Chinese massage therapy on the basis of the control group, or simply given Chinese massage therapy. Each group of selected Chinese massage techniques may be different.

#### 2.3.4 Types of outcome measures

Primary outcome measure: overall clinical response rate, breast pain score. Secondary outcome measures: milk secretion, temperature, mass size and time to resolution, White blood cell count, C-reactive protein, and the incidence of adverse reactions.

### 2.4 Data sources

#### 2.4.1 Electronic searches

Our study will be conducted through computer retrieval of eight electronic databases with a deadline of November 30, 2021. The main search terms are expanded to meet the specific requirements of the Chinese database. All literatures in English databases were searched by MeSH subject words plus free words. PubMed retrieval strategy was demonstrated in the retrieval process, as shown in Table 1.

**Table 1:** Search strategy of PubMed.

Number	Search item
# 1	Mastitis [MESH]
#2	Mastitis[Title/Abstract] OR Mammitis [Title/Abstract] OR Mastadenitis [Title/Abstract] OR Acute Mastitis [Title/Abstract] OR Acute Mammitis [Title/Abstract] OR Inflammation of Mammary Gland [Title/Abstract]
#3	# 1 OR #2
#4	Massage [MESH]
#5	Massage [Title/Abstract] OR Zone Therapy [Title/Abstract] OR Zone Therapies [Title/Abstract] OR Therapy, Zone [Title/Abstract] OR Therapies, Zone [Title/Abstract] OR Massage Therapy [Title/Abstract] OR Massage Therapies [Title/Abstract] OR Therapy, Massage [Title/Abstract] OR Therapies, Massage [Title/Abstract] OR Tuina [Title/Abstract]
#6	#4 OR #5
#7	Randomized controlled trial [Publication Type] OR randomized [Title/Abstract] OR randomly [Title/Abstract]
#8	#3 AND #6 AND #7

#### 2.4.2 Searching for other resources

Books, potential grey literature, conference papers and reports and other relevant materials will be manually searched.

### 2.5 Data collection and analysis

#### 2.5.1 Selection of studies

**Figure 1:**
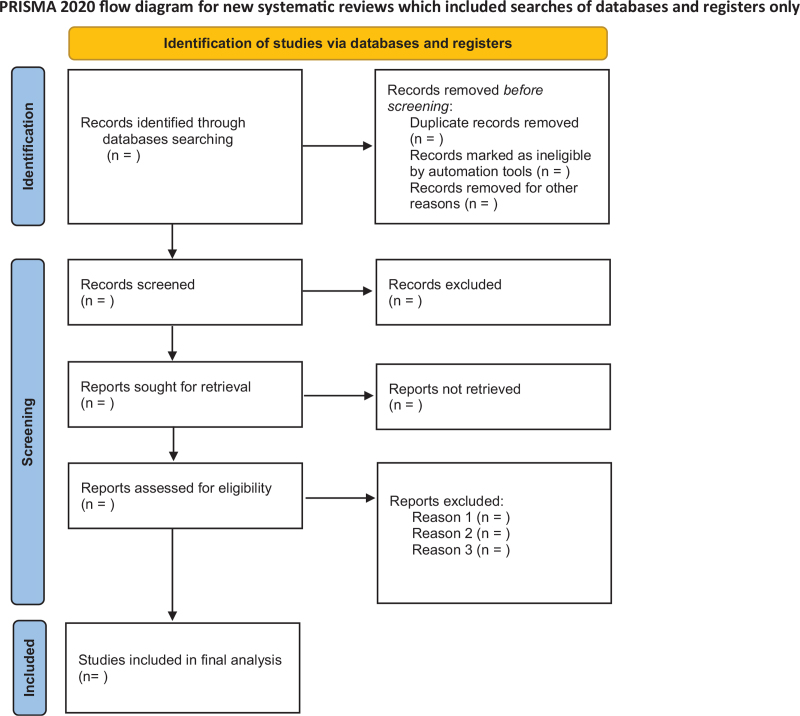
The flowchart of the screening process.

All literature searched from the database is entered into EndNote X9 software for screening. After the same literature was excluded, two researchers (Longsheng Ren and Ruiying Guo) further read the title and abstract of the literature to exclude the literature that did not meet the requirements. The selected literature was comprehensively read to determine the final literature to be included in the study. Any differences arising during the screening process will be discussed with a third party (Can Lyu) and the final decision will be made. The process of literature screening is shown in Figure 1.

#### 2.5.2 Data extraction and management

Two researchers (Longsheng Ren and Ruiying Guo) independently extracted data information. Any disagreement will be discussed and decided with third party (Can Lyu). Information extracted included: first author, year of publication, age, duration of disease, duration of treatment, outcome indicators, sample size, interventions, adverse reactions, etc. The extracted information was compiled into Excel tables for data collection. If the data information in the literature is incomplete or inconclusive, we will attempt to complete the information by contacting the original author by email or telephone. If experimental data cannot be obtained by contacting the authors, we will exclude the study.

#### 2.5.3 Risk of bias assessment

Two researchers (Longsheng Ren and Ruiying Guo) independently evaluated the quality of the included literature according to the RCT risk assessment tool in the Cochrane Evaluation Handbook 5.4. The main evaluation items include random sequence generation, assignment concealment, blindness, selective reporting results, incomplete results data, and other biases. Each assessment item is divided into three sections: “low risk”, “unclear risk” and “high risk”. All differences that exist will be resolved through our discussions with third parties (Can Lyu).

#### 2.5.4 Data analysis

Meta-analysis of the data from the included studies will be performed by using the data processing software RevMan 5.4 of the Cochrane Collaboration Network. The relative risk (RR) of the 95% confidence interval (CI) will be calculated using dichotomous variables, and the mean difference (MD) of the 95% confidence interval (CI) will be calculated using continuous variables. *P* < .05 was considered statistically significant. *P* values and I^2^ values will be used to test for heterogeneity in the included study literature. I^2^ < 50% was taken as the standard for no heterogeneity or small heterogeneity, and fixed effect model was used for analysis. If heterogeneity exists or is large, I^2^ > 50% is taken as the standard, and random effect model is used for analysis. If heterogeneity exists, sensitivity analysis or subgroup analysis will be performed to determine the source of heterogeneity. Descriptive analysis will be used when meta-analysis is not possible.

#### 2.5.5 Subgroup analysis

Factors such as age, course of disease, duration of treatment and different interventions will be analyzed by subgroup to explore the source of heterogeneity.

#### 2.5.6 Sensitivity analysis

Sensitivity analysis will be used to explore the significant heterogeneity in the studies and identify the sources of heterogeneity.

#### 2.5.7 Assessment of reporting biases

If more than 10 studies were included in the meta-analysis, we will use Revman5.4 software to draw funnel plot to analyze whether there is publication bias in this study and analyze the results.

#### 2.5.8 Grading the quality of evidence

In this study, the Grading of Recommendations Assessment, Development, and Evaluation (grade) method will be used to evaluate the quality of outcome evidence. The grade of evidence quality will be divided into four categories: very low quality, low quality, medium quality and high quality.

## 3 Discussion

The incidence of AM is increasing. According to a survey, the probability of nursing women suffering from AM is as high as 33%, and the probability of mammary abscess occurring if the treatment is improper or delayed is 3–11%, and even sepsis and other serious problems will occur.^[7,16]^ In modern medicine, the effect of antibiotic treatment for early AM is relatively limited.^[17]^ On the one hand, the curative effect will be reduced due to the development of drug resistance in patients. On the other hand, patients will have anxiety due to the use of antibiotics to affect breastfeeding, which has a great impact on the physical and psychological of patients. In order to solve the problem of AM treatment is not enough and improve the quality of life of patients and infants, we believe that AM can be treated by Chinese massage. It has been found that the duration and treatment effect of AM can be improved by promoting breast empties.^[18,19]^ Chinese massage has the function of dredging mammary duct and promoting the emptying of mammary gland. It can improve the circulation of breast local blood and lymph fluid, promote the absorption of oedema and inflammatory products, reduce the pressure inside the mammary gland. At the same time, the application of Chinese massage can also reduce patients’ concerns about the use of drugs affecting lactation, and has a significant effect on the treatment of AM.

However, the Chinese massage for AM lacks the support of evidence-based medicine. Therefore, our study will conduct a systematic evaluation and meta-analysis on the effectiveness and safety of Chinese massage treatment of AM, aiming to provide a stronger evidence-based basis for clinical practice.

## Author contributions

**Conceptualization:** Qiang Wang, Jie Zhang.

**Data curation:** Longsheng Ren, Ruiying Guo, Can Lyu.

**Formal analysis:** Longsheng Ren, Ruiying Guo.

**Investigation:** Longsheng Ren, Ruiying Guo, Can Lyu.

**Methodology:** Longsheng Ren, Danyang Zhao, Zhihao Dong.

**Project administration:** Qiang Wang, Jie Zhang.

**Resources:** Longsheng Ren.

**Software:** Longsheng Ren, Ruiying Guo, Can Lyu.

**Supervision:** Qiang Wang.

**Validation:** Zhihao Dong, Zenglin He.

**Visualization:** Longsheng Ren.

**Writing – original draft:** Longsheng Ren, Can Lyu, Qiang Wang.

**Writing – review & editing:** Longsheng Ren, Qiang Wang.
